# Validation of the modified Japanese Triage and Acuity Scale-based triage system emphasizing the physiologic variables or mechanism of injuries

**DOI:** 10.1186/s12245-015-0097-9

**Published:** 2016-01-25

**Authors:** Hiraku Funakoshi, Takashi Shiga, Yosuke Homma, Yoshiyuki Nakashima, Jin Takahashi, Hiroshi Kamura, Masatomi Ikusaka

**Affiliations:** Department of Emergency Medicine, Tokyobay Urayasu Ichikawa Medical Center, 3-4-32 Todaijima, Urayasu-shi, Chiba 279-0001 Japan; Department of General Medicine, Chiba University Hospital, 1-8-1 Inohana Chuo-ku, Chiba-shi, Chiba 260-8677 Japan

**Keywords:** Triage, Patient safety, Canadian Triage and Acuity Scale, Japanese Triage and Acuity Scale

## Abstract

**Background:**

The Canadian Triage and Acuity Scale is a valid triage system. The system was translated and implemented in the Japanese emergency departments (EDs) from 2012. This system was named the Japanese Triage and Acuity Scale; however, the validation studies of the Japanese Triage and Acuity Scale have been limited. In addition, for a patient with multiple complaints, it could become challenging, due to its requirement of a single complaint. Therefore, we hypothesized that a modified version of the Japanese Triage and Acuity Scale using first-order modifiers without chief complaint detection is accurate.

**Methods:**

A retrospective cohort study evaluated a correlation between the modified triage scale level and outcomes of all adult emergency department patients at a Japanese hospital.

Construct validity of the modified triage scale level was assessed based on comparisons of total admission rate (including hospitalizations, emergency department deaths) and length of stay between triage levels.

**Results:**

The distributions of five levels of the triage scale (level 1 is the most urgent) among the 17,121 cases are as follows: 1:451, 2:1148, 3:7703, 4:7652, and 5:167. Total admission rates by each level were 1:89.8, 2:68.2, 3:26.4, 4:6.6, and 5:0.6 %, which progressively increased from level 5 to 1 and were significant (*p* < 0.01). Compared with patients in level 3, the odds of total admission rates were 14.4, 5.1, 0.27, and 0.030 for the patients in levels 1, 2, 4, and 5. The length of stay was longer in the patients with the more urgent levels except for those with level 1.

**Conclusions:**

The modified version of the Japanese Triage and Acuity Scale is a valid predictor of total admission and length of stay and may enable the nurses to triage patients without detecting the chief complaints.

## Background

In the emergency department (ED), a reliable triage system is essential to assess the patients’ severity of injury or illness within a short time after their arrival, to assign priorities and to transfer each patient to the appropriate place for treatment [1]. The Canadian Triage and Acuity Scale (CTAS) is one of the most widely accepted web-based triage systems that has shown high validity and reliability in many research studies in various countries [2–6].

In April 2012, the Japanese Society for Emergency Medicine translated and implemented the CTAS in the Japanese EDs. This system was named the Japanese Triage and Acuity Scale (JTAS). The JTAS has been well accepted in many EDs in Japan. However, the validation studies of the JTAS have been limited, and it has several issues to solve [7].

First, ED overcrowding has been recognized as a major concern leading to a decrease in the quality of care in the EDs. Indeed, two studies have demonstrated an association between ED crowding and increased mortality [8, 9]. A triage system should not only be valid and reliable but also prompt. From this point of view, there are challenges associated with using the JTAS for triage staff, because the system requires the evaluation of a single chief complaint, even though many patients, especially older patients, have multiple complaints. When a patient is triaged based on the JTAS, the triage nurse initially identifies the patient’s single chief complaint. In the second step, the triage nurse determines the triage level, mainly relying on first-order modifiers non-specific to each complaint. Most of the first-order modifiers are common among chief complaints.

Second, the JTAS is notable for its requirement of web connectivity. Due to the limitations of the web connectivity in the Japanese healthcare environment because of guidelines from the Japanese government regarding personal information protection, a triage system based on the JTAS without a web connectivity requirement is beneficial for many Japanese institutions.

The Emergency Severity Index (ESI) is a well-validated triage system that does not require a single chief complaint and is also well validated. Therefore, we examined our hypothesis that the triage level would be accurate when the modifiers were used from the beginning of triage without detecting a single chief complaint.

This is one of the few studies to evaluate the feasibility and validity of a JTAS-based triage system in Japan.

## Methods

### Study design, setting, and sample

We performed a retrospective cohort study of all the adult ED patients who presented to our emergency department (which has 18 ED patient care beds) and were seen by the emergency physicians from April 1, 2013 to March 31, 2014. The study was reviewed and approved by the local Institutional Review Board. The clinical practice committee in our hospital approved implementing this new system because we examined inter-rater reliability of this JTAS-based triage system and presented at the Japanese Society of Emergency Medicine scientific assembly prior to the implementation, and there is no triage system with robust scientific validation in Japan. It was performed at Tokyo Bay Urayasu Ichikawa Medical Center, a 344-bed urban acute care community hospital with an annual ED census of 31,793, in eastern Tokyo, Japan. This is a regional trauma center and designated stroke/cardiovascular center with 24-h capability of percutaneous coronary artery intervention and infusion of t-PA.

All of the adult (18 years old or above) patients treated during the study period seen by board-certified emergency physicians were eligible for inclusion. We excluded all pediatric patients under 18 years of age and adult patients who arrived at the ED to be seen directly by the specialist.

### Study protocol

Table 1 shows our triage system.

**Table 1:** The mJTAS triage scale. The triage nurse checks each domain and determines the patient’s triage level based on the domain with the most significant triage level

Triage level	Resuscitation	Emergency	Urgency	Low urgency	Non-urgency
Consciousness	GCS <9	GCS 10–13	GCS 14	Alert	
Breathing	SpO_2_ <90 % or speak only single word or upper airway obstruction	SpO_2_ <92 % or labored breathing	SpO_2_ <94 % or respiratory rate >22	SpO_2_ >95 % and breathing normally	
Circulation	Shock (systolic blood pressure under 80 mmHg or shock index (systolic blood pressure/heart rate) <1) or weak pulse	Diaphoresis or tachycardia (over 120 beats per minute) or history of orthostatic hypotension	Apparently abnormal blood pressure (systolic blood pressure >160 or <100 mmHg)	Normal blood pressure	
Thermal status		SIRS criteria more than 3/4, including a fever higher than 38.5° or known to have neutropenia or a steroid user	SIRS criteria 2/4, including a fever higher than 38.5°	Fever only	
Pain			Pain scale >8/10	Pain scale 4–8/10	
Bleeding		Active bleeding from head, neck, or trunk or accompanied with an open fracture	Epistaxis or active bleeding from oral cavity	Stopped bleeding	
Mechanism of injury		Ejected from a vehicle over 40 km/h or pedestrian hit by a vehicle or Fall from over 6 m or penetration injury	Motor vehicle accidents which do not meet the description within the left column	Bicycle accidents	
Special situation	Cardiopulmonary arrest	Acute chest pain or hemiparesis			Stable symptom(s) over 8 weeks

*SIRS* systemic inflammatory response syndrome

We created the modified Japanese Triage and Acuity Scale (mJTAS) according to the original JTAS first-order modifiers and used the mJTAS for the ED referred to in the paper. The mJTAS system comprises eight objective domains, which are the mental status, respiratory status, circulatory status, thermal status, pain status, hemorrhagic status, mechanism of injuries, and Special situation in case of trauma. Each domain has four to five triage levels. The only exceptions to this system are chest pain and hemiparesis, which are always assigned to be level 2 or less. This is because myocardial infarction, cerebral infarction, and cerebral hemorrhage require early intervention, even though patients with those conditions could have normal vital signs. The triage nurse checks each domain and determines the patient triage level based on the domain with the most significant triage level.

We present a typical flow of the mJTAS below. When a 50-year-old male presented with shortness of breath and palpitation to our hospital, our triage nurse measured his vital signs regardless of his complaint. His level of consciousness was E4V5M6 in the Glasgow Coma Scale. His vital signs were respiratory rate 22/min, blood pressure 110/70 mmHg, heart rate 100/min, O_2_ saturation 89 %, and temperature 39.0 °C. According to Table 1, level of consciousness was assigned to low-urgency level, breathing was assigned to resuscitation level, circulation was assigned to low-urgency level, and temperature was assigned to emergency level. We determine the triage level based on the highest triage level obtained from all domains. Therefore, his triage level was assigned to the resuscitation level in this case.

The implementation of the mJTAS at the study site began in July 21, 2012. Since then, the mJTAS has been used for every ED patient with no exceptions. There were no occasions that required a change in the mJTAS domains or triage levels or variables. Level 1 patients are the most critically ill and need immediate resuscitation.

The ED nurses were registered nurses who had official training on the measurement of vital signs and/or the level of consciousness, and had standardized training about the mJTAS system prior to the study period.

### Study variables

The variables assessed for a correlation with the patient triage level were the total admission rate (TAR; including hospitalizations, emergency department (ED) deaths, and transportation to another hospital for admission) and ED length of stay (LOS). The ED LOS was defined as the time in minutes from registration to discharge or admission [10]. These variables were abstracted from electronic medical records. The primary investigator and co-investigators have independently assessed the reliability of the data entry.

### Data analysis

The primary null hypothesis of this study was that there was no correlation between the mJTAS level and the TAR. The chi-square test was performed for categorical variables. In addition, a multivariate logistic regression analysis adjusted for the patient age, sex, and ambulance use was employed to analyze the relationships between the triage level and the rate of total admission. *p* values were based on a significance level of 0.05.

For the LOS, the non-parametric Kruskal-Wallis test was used to examine the differences in the ED LOS among triage levels. The raw data for the LOS were reported as medians and interquartile ranges (IQR; 0.25, 0.75). We used a Bonferroni correction to account for the fact that we employed multiple statistical testing; a *p* value of 0.005 was used to signify statistical significance.

The 95 % confidence intervals (CI) were reported for every result. Statistical calculations were conducted using the STATA (Version 12) software package.

## Results

### Patient characteristics and triage level

During the study period, 31,793 patients presented to our emergency department. Out of the eligible patients, 13,485 patients who were under 18 years of age, and 425 patients who arrived at the ED to be seen directly by the specialist were excluded. In addition, 762 were excluded from the data analysis due to missing data (514 patients, 2.9 %) or because the patients were presumed to be outliers due to registration errors (248 patients, 1.4 %; a LOS >720 min or LOS <10 min). The number of final study samples was 17,121. Of these cases, the mean age was 50.6 years of age (± standard deviation (SD) ±20.7), 8858 (51.7 %) were male, and 6,759 (39.4 %) were transported by ambulance. The disposition included 13,389 (78.2 %) discharges, 3481 (20.3 %) admissions, 160 (0.9 %) cases of transportation to another hospital for admission, and 91 (0.5 %) ED deaths. The median LOS was 149.3 (IQR; 10, 717) min.

The distributions of the triage levels among the 17,121 cases were as follows: level 1, 451 (2.6 %); level 2, 1148 (6.7 %); level 3, 7703 (45.0 %); level 4, 7652 (44.7 %); and level 5, 167 (1.0 %). Table 2 shows the patient characteristics by each triage level.

**Table 2:** The baseline characteristics stratified by the mJTAS level

	Resuscitation	Emergency	Urgency	Low urgency	Non-urgency
Number of cases	451	1148	7703	7652	167
Mean age (±SD)	68.8 (±19.7)	61.1 (±20.2)	52.8 (±20.7)	45.9 (±19.4)	46.9 (±19.4)
Male (%)	250 (55.4 %)	671 (58.5 %)	4006 (53.0 %)	3755 (49.1 %)	96 (57.5 %)
Ambulance use (%)	399 (88.5 %)	766 (66.7 %)	3602 (46.8 %)	1988 (26.0 %)	4 (2.4 %)

*SD* standard deviation

### Disposition

Table 3 summarizes the details of the disposition by each triage level.

**Table 3:** The details of the disposition stratified by the mJTAS level

	Resuscitation	Emergency	Urgency	Low urgency	Non-urgency
	(*N* = 451)	(*N* = 1148)	(*N* = 7703)	(*N* = 7652)	(*N* = 167)
Discharge (%)	46 (10.2 %)	365 (31.8 %)	5668 (73.6 %)	7144 (93.4 %)	166 (99.4 %)
Admission (%)	308 (68.3 %)	752 (65.5 %)	1931 (25.1 %)	490 (6.4 %)	1 (0.6 %)
Transportation to another hospital for admission (%)	7 (1.6 %)	30 (2.6 %)	104 (1.4 %)	18 (0.2 %)	0 (0 %)
Death in ED (%)	90 (20.0 %)	1 (0.1 %)	0 (0 %)	0 (0 %)	0 (0 %)

The TARs by each triage level were as follows: level 1, 89.8 %; level 2, 68.2 %; level 3, 26.4 %; level 4, 6.6 %; and level 5, 0.6 %. The TAR progressively increased from triage level 5 to level 1, and this increase was statistically significant (c2 = 3845; df 4; *p* < 0.001).

To analyze the relationship between the mJTAS triage level and the TARs, we developed a logistic regression model. The mJTAS level 3 was used as a reference marker for this evaluation because this was the most common score. Among the triage levels, there were statistically significant differences in that the higher levels were associated with a higher proportion of the total admission, as well as an older age (odds ratio 1.04 95 % confidence interval (CI) 1.04–1.04), female gender (odds ratio 0.80 95 % CI 0.73–0.88), ambulance usage (odds ratio 2.71 95 % CI 2.47–2.98), and LOS (odds ratio 1.00 95 % CI 1.00–1.00). Compared with patients in the mJTAS level 3, the odds ratio for the total admission was 15.1 (95 % CI 10.9–20.9), 5.2 (95 % CI 4.5–6.0), 0.27 (95 % CI 0.25–0.31), and 0.030 (95 % CI 0.005–0.24) for the patients in the mJTAS levels 1, 2, 4, and 5, respectively. The area under the receiver operating characteristic curve for this model was 0.86.

### Length of stay

**Fig. 1:**
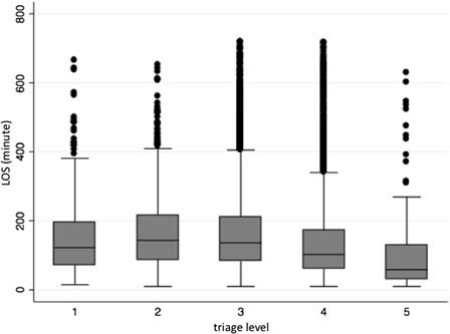
The length of stay in the ED stratified by the mJTAS level. The *box plots* indicate the median (*horizontal line*); the interquartile range (*box*), not farther away than 1.5 times the interquartile range from the first and third quartiles (*whiskers*); and the values that are not in the range of *whiskers* and not considered outliers (*dots*)

The LOS in the ED after registration was longer in the patients with more urgent triage levels, except for the patients with triage level 1 (resuscitation). Figure 1 summarizes the LOS by each triage level. The Kruskal-Wallis test demonstrated statistically significant differences between the LOS based on the triage level (*p* < 0.001).

## Discussion

Our study demonstrated that the triage level assigned by the triage nurses using the mJTAS was strongly associated with the TAR and an increased length of stay in the ED. As previously mentioned, we demonstrated that the mJTAS had high inter-rater reliability with an intraclass correlation coefficient of 0.77 (Haruka T: Reliability of a CTAS base triage system, unpublished). The reasons for this high inter-rater reliability could be that in this triage system, almost all factors that determine the triage level are objective, such as vital signs or the level of consciousness, and this scale does not require the detection of chief complaints.

The TAR has traditionally been considered an important indicator of severity. Our study confirmed that there is a strong relationship between the mJTAS level and the TAR. However, an admission to hospital is not always a good surrogate marker for severity. For example, patients with a hip fracture are often admitted to the hospital, but their pain status is not significant unless the hip joint is moved, and they often have stable vital signs. In contrast, patients with alcohol intoxication rarely require admission to hospital, although they have a higher level of urgency because they tend to deteriorate quickly or have a low level of consciousness. There are numerous clinical scenarios such as these cases, in which the need for urgent medical assessment may not correlate with hospital admission. However, many studies have adopted an admission to hospital as a surrogate marker [3–6, 11–15].

The LOS of patients in the mJTAS level 1 cohort was less than that of patients in the mJTAS level 2 or 3 cohorts. Critically ill patients are associated with relatively simple decisions regarding disposition and are usually transferred to the intensive care unit (ICU). Patients with cardiopulmonary arrest typically receive cardiopulmonary resuscitation for less than 30 min, and once they are resuscitated, they are immediately transported to the ICU. These results are very similar to those described by Dong [2] who reported that the LOSs were 197 min in the resuscitation category, 351 min in the emergency category, 309 min in the urgent category, 206 min in the low-urgent category and 130 min in the non-urgent category in patients who were triaged using the CTAS in their ED. Gravel [11], Elshove-Bolk [16], and Storm-Versloot [10] also reported similar results using different triage systems.

Based on our data using the mJTAS, it could be possible for the hospital to anticipate the necessary inpatient resources because the mJTAS levels have accurate correlations with the TAR and ED LOS.

As demonstrated, the results of our study are similar to the previous literature on the original version of the CTAS [2–6]. It means that we might be able to bypass the first step of the CTAS, which is the identification of the chief complaint of the patient, thus leading to faster triage and avoiding issues in patients with multiple complaints. In addition, our study results showed comparable validity with a web-based triage system, which may encourage many Japanese hospitals with limited web connectivity due to personal information protection regulation to use a scientifically validated triage system.

### Limitation

Although, the population pyramid of the Urayasu Ichikawa Area is quite similar to the national population pyramid, one of the limitations of our study design was that this study was conducted at the ED of a single community hospital in Japan. This may limit the generalizability of the results, and a larger multi-center study would be needed to confirm the present findings. Second, the emergency physicians at the study site were not blinded to the triage level, which may have influenced their decision on the disposition. Third, the rate of mistriage is not known. Mistriage could cause data contamination and result in an underestimation or overestimation of the outcome differences. Fourth, our study showed comparable correlation between triage levels and of patients’ outcomes such as total admission rate or length of stay with the previous reports of the triage system. However, due to different patient populations between the studies, our study has limitations with regard to its generalizability.

## Conclusions

The modified JTAS triage system is a valid predictor of hospital admission and the ED LOS in the Japanese ED population. The mJTAS may enable the nurses to triage patients without detecting the chief complaints. In addition, the mJTAS would be useful for resource-limited settings, such as in areas where an Internet connection is unavailable.

## Abbreviations

CI: confidence intervals
CTAS: Canadian Triage and Acuity Scale
ED: emergency department
ICU: intensive care unit
IQR: interquartile ranges
JTAS: Japanese Triage and Acuity Scale
LOS: length of stay
mJTAS: modified JTAS
SD: standard deviation
TAR: total admission rate
